# Perceived Physical Competence Predicts Gains in Children’s Locomotor but Not Ball Skills across an Intervention

**DOI:** 10.3390/ijerph18115990

**Published:** 2021-06-03

**Authors:** Kara K. Palmer, Michael A. Nunu, Katherine Q. Scott-Andrews, Leah E. Robinson

**Affiliations:** School of Kinesiology, University of Michigan, Ann Arbor, MI 48109, USA; palmerka@umich.edu (K.K.P.); minunu@umich.edu (M.A.N.); katieqa@umich.edu (K.Q.S.-A.)

**Keywords:** perceived competence, motor competence, self-perceptions, TGMD, pediatrics

## Abstract

The purpose of this pre/post experimental study was to examine if children’s perceived physical competence predicted changes in motor skills across an intervention. Sixty-seven children (Mage = 53.2 ± 3.7 months) participated in a 16-week, mastery-climate motor skill intervention. Perceived physical competence was assessed before the intervention using the physical competence subscale of the Pictorial Scale of Perceived Competence and Social Acceptance for Young Children. Motor skills were assessed using the Test of Gross Motor Development-3rd Edition before and after the intervention. Results revealed that controlling for pretest skills, perceived physical competence significantly predicted posttest locomotor (*p* < 0.05) and total skills (*p* < 0.05) but did not predict posttest ball skills (*p* > 0.05). These results indicate that perceived physical competence may be a significant factor that predicts children’s gains in locomotor or total skills, but not ball skills, across an intervention.

## 1. Introduction

Gross motor skills are an important component of current and future health in children [1]. These skills are large muscle movements needed to maintain posture and propel or manipulate objects (ball skills) or the body (locomotor skills) through space [2]. Unfortunately, many children do not exhibit proficiency in gross motor skills [3], and children fail to learn these skills in the absence of targeted instruction or intervention [4,5,6]. Children with poor gross motor skills may fall into sedentary behaviors or negative trajectories of health [1,7]. Therefore, it is vital to teach, practice, and reinforce these skills in children for their current and long-term health [8,9].

Gross motor skill interventions, intentional instruction or programs specifically designed to teach motor skills, are an effective approach to teach these skills to young children [4,5,6]. While the evidence supporting the effectiveness of gross motor skill interventions is encouraging, there is a need for a deeper understanding of how individual constraints might be contributing to changes in children’s skills across an intervention. Individual constraints are intrinsic to the performer and can be structural (e.g., the physical structure of the individual) or functional (e.g., internal factors such as motivation or self-perceptions [10]). Each child brings their own unique individual constraints to an intervention setting, and these constraints may be important factors to consider regarding the effectiveness of the intervention for that child. For example, research supports that a child’s individual constraint of motor skills at the start of an intervention relates to changes in motor skills across the intervention so that children with better initial skills have smaller gains in motor skills across the program [11]. Therefore, evidence supports that individual constraints at the start of an intervention may be important to consider when examining intervention efficacy.

One individual constraint often included in conceptual models of motor skills and health is perceived competence or an individual’s awareness and belief about their abilities [1,7,12]. Young children typically cannot differentiate between ability and effort, so perceived competence at a young age is likely attributed to effort and practice of tasks and not actual skills or abilities [8,13,14]. Perceived physical competence (PPC) is a child’s belief about their ability to move [8] and is associated with children’s motor competence [1,15] and physical activity [16,17]. Perceived competence is also associated with children’s and youth’s motivation to engage in physical education [18] and physical activity [19]. PPC may be an important individual constraint to consider in other movement environments, such as gross motor skill interventions. This individual factor may be important in interventions in early childhood, when children are unable to assess their own abilities accurately and when self-perceptions are generally high [8,13,14].

Due to the established link between perceived competence and motivation, PPC might be critical to consider in intervention climates specifically designed using motivational theories such as achievement goal theory. Achievement goal theory represents how children approach, engage, and respond to educational or learning activities [20,21,22,23] and is grounded in the belief that children are innately motivated to learn and explore their environment [24]. This theory states that individuals approach learning in two ways: with a mastery- or performance orientation [20,21,22,23]. A mastery-oriented approach to learning means an individual engages in learning for learning’s sake, tracks progress based on self-referenced norms, and equates effort with learning [20,21,22,23]. In contrast, a performance-oriented approach to learning means the individual engages in learning to demonstrate competence, tracks progress based on comparative norms, and equates effort with an inability to show learning [20,21,22,23]. Adopting a mastery-oriented approach to learning is associated with learning benefits, including intrinsic interest in learning [25,26], positive attitudes towards learning [25,27], and persistence in the face of difficulty. Learning environments can be manipulated to foster a mastery-oriented approach to learning [28]. Research supports that mastery-oriented climates are an effective approach in developing and implementing gross motor skill interventions for children, and these interventions have immediate and sustained positive effects on children’s gross motor skills [29,30]. These interventions also improve children’s PPC, whereas low-autonomy climates or learning environments not designed to support a mastery-oriented approach to learning are not effective at supporting PPC [31].

In conclusion, there are theoretical and research-driven links between PPC and children’s motor skills in an intervention context, particularly interventions implementing mastery climates using achievement goal theory. More research is needed to understand if a child’s PPC at the beginning of a mastery-oriented intervention predicts his/her gains in motor skills across the intervention. The objective of this study was to examine if children’s PPC at the start of a mastery-oriented gross motor skill intervention predicted change in motor skills across the intervention controlling for pretest motor skills and sex. We hypothesized that children who have greater PPC at the beginning of the intervention would have more significant changes in motor skills across the intervention.

## 2. Materials and Methods

### 2.1. Study Design

This research used a pre/post within-subjects experimental design.

### 2.2. Participants

The participants were 71 children (Mage = 53.2 ± 3.7 months, 29 boys) from two Head Start Centers in a small city in the United States. Parents were eligible to enroll their children in a Head Start program if their annual income was at or below the federal poverty line (i.e., <$26,200 USD for a family of 4). Children between 3.5–5 years of age with no cognitive or developmental disabilities on record with the school were invited to participate in the study.

### 2.3. Measures

#### 2.3.1. Perceived Competence

Perceived competence was assessed as PPC using the Pictorial Scale of Perceived Competence and Social Acceptance for Young Children (PSPCSA; ref. [8]). The scale is a valid and reliable pictorial scale assessing perceived competence for children ages 4–7 years [8]. The scale consists of four subscales: cognitive competence, physical competence, peer acceptance, and maternal acceptance. In alignment with the objective of this study, only the physical competence subscale was used to assess PPC. The six items on this subscale include swinging, climbing, skipping, running, tying shoes, and hopping. Previous research has used this subscale to assess PPC in similar samples [31,32,33].

For the subscale, children were presented with two images of children of the same gender completing each skill: one image was of a skilled performance and one of an unskilled performance. The child was read a statement describing the performance in each image and then was asked to indicate which of the two images is like them. After this, the child was asked to describe to what extent they look like the image. Each response corresponded with a point value ranging from 1–4 where a 4 represents the most competent and a 1 represents the least competent. For the image of the unskilled performance, children were asked, “are you sort of good at this skill (2) or not too good at this skill (1)?” For the image of the skilled performance, children were asked, “are you really good at this skill (4), or pretty good at this skill (3)?” In the current study, scores across all six items (α = 0.54) at pretest were averaged, and the average subscale score (possible range: 1–4) was used in analyses.

#### 2.3.2. Motor Skills

Motor skills were assessed using the Test of Gross Motor Development, 3rd edition (TGMD-3; ref. [34]). The TGMD-3 is a valid and reliable process-oriented assessment used to measure motor skills in children ages 3 through 10 years. The TGMD-3 measures performance on seven ball skills (1-hand strike, 2-hand strike, dribble, toss, kick, throw, and catch) and six locomotor skills (run, gallop, hop, skip, slide, and jump). Each motor skill is divided into 3–5 predetermined specific performance criteria, and the child receives a 1 if they perform a skill criterion correctly and a 0 if they fail to perform the criterion. Children performed three skill trials: one practice trial and two scored trials. Children received a digital demonstration of the skill before the practice trial [35]. If a child did not understand the motor skill during the practice trial, the child was given a second live demonstration of the skill. Identical verbal instructions were provided in both the digital and live demonstrations. The child then completed two test trials, which were filmed so skill performances could be coded remotely. Total raw scores for the locomotor subscale (0–44), ball skill subscale (0–56), and total TGMD (0–100) were calculated. Raw scores were used in analyses. Two coders coded all the data. The primary coder for this research had a previously established inter-rater reliability of >95% with three external motor experts and established reliability with the TGMD-3 online training (https://sites.google.com/a/umich.edu/tgmd-3/reliability-videos) (accessed on 5 August 2020). A second, blinded, expert coder cross-coded 25% of the sample. The two coders demonstrated high inter-rater reliability (intra-class correlation = 0.88 locomotor, 0.93 ball FMS, 0.96 total).

### 2.4. Intervention

All children in this study participated in the Children’s Health Activity Motor Program (CHAMP; ref. [9,32,36,37]). CHAMP is a motor skill intervention grounded in achievement goal theory [20,25]. CHAMP is effective in improving children’s motor skills [9,35]. Two trained instructors with graduate degrees in motor development implemented CHAMP. The lead instructor had five years of previous experience with CHAMP, and the secondary instructor had a degree in physical education. Each CHAMP session lasted 45 min and consisted of the following components: (1) a 2-min warm-up activity; (2) 3–4 min of motor skill instruction; (3) 32–35 min of autonomy-based motor skill engagement, in which children were free to choose where and how long to engage in each motor skill activity; and (4) 2–3 min of a closure activity. CHAMP replaced the children’s standard outdoor free play 3 days a week for 16 weeks (48 sessions, total dose = 2160 min).

### 2.5. Procedures

Data for this secondary analysis came from the Promoting Activity and Trajectories of Health (PATH) study (Registered Clinical Trial Number: NCT03189862, www.clinicaltrials.gov; ref. [37]) (accessed on 1 May 2021). The PATH project was approved by the Institutional Review Board at the University of Michigan (HUM00133319), and study procedures have been fully described and published elsewhere [37]. For this secondary data analysis, only the children who were randomly selected to participate in the intervention during the first year of the study were included. Seventy-one children were originally included in the sample, but four did not complete the intervention and were excluded from this analysis leaving 67 children in the final sample. Sex and race were reported on the parental report forms. Children completed both PPC and TGMD-3 assessments at pretest (October) and posttest (April). Children completed the PPC 24-h before completing the TGMD-3. Between pretest and posttest, all children participated in the CHAMP intervention 3 days a week for 16 weeks.

### 2.6. Data Analyses

Descriptive (i.e., mean and standard deviation) and normality statistics (i.e., skewness and kurtosis) were calculated for all measures to check the distribution of the data. Since data were normally distributed, the efficacy of the CHAMP intervention for improving motor skills was examined through a series of paired *t*-tests. Stepwise linear regression models were fit to examine if pretest skills, PPC, and sex predicted posttest motor skills. A series of models were fit for locomotor skills, ball skills, and total skills separately. All analyses were completed in SPSS 26 (IBM Corp, Armonk, NY, USA), and alpha levels were set to 0.05 a priori.

## 3. Results

### 3.1. Intervention Efficacy

Compared with pretest, children had higher posttest locomotor, ball skills, and total skills (all, *p*’s <0.001; see Table 1).

### 3.2. Predictors of Posttest Motor Skills

Children had average PPC scores of 3.20 ± 0.58 at pretest. Controlling for pretest and sex, PPC significantly predicted posttest locomotor (b = 3.20, 1.23, SE = 1.23, *p* = 0.012) and total TGMD raw scores (b = 4.42, SE = 1.96, *p* = 0.028; see Table 2). PPC did not predict posttest ball skill score. Controlling for pretest and PPC, sex moderated post-test ball skill scores (b = 4.63, SE = 1.50, *p* < 0.001; see Table 2), indicating that boys had higher posttest ball skills compared with girls.

## 4. Discussion

Individual-level characteristics may be important to consider when examining children’s skill changes across a gross motor skill intervention [10]. Perceived competence, specifically PPC, is an individual constraint related to motor skills [38] and physical activity [16,17]. Young children demonstrate inflated self-perceptions that may encourage them to be active and practice these skills [7]. Therefore, PPC may be an important individual-level factor to consider regarding intervention effects in young children. The purpose of this study was to examine if children’s PPC at the start of an intervention predicted changes in motor skills across an intervention. We hypothesized that children who have greater PPC at the beginning of a motor skill intervention would have more significant changes in motor skills across the intervention.

The results partially supported our hypothesis. Controlling for pretest, PPC significantly predictor change in locomotor skills and total skills but not ball skills across the CHAMP intervention. While it is unclear why PPC only predicted some and not all skills, this differentiating result between skill types may be due to the measure of PPC itself. The measure utilized to assess PPC was the physical subset of the PSPCSA. This scale measures PPC across six motor tasks: swinging, climbing, skipping, running, tying shoes, and hopping [8]. Of these six tasks, five are gross motor tasks centered on play (swinging and climbing) or locomotion (skipping, running, hopping). No ball skills are included in the PPC subscale. This lack of ball skills may result in more perceived locomotor competence verse global or ball skill competence. Results may have differed if we had assessed perceived competence using a different measure that included locomotor and ball skills such as the Pictorial Scale of Perceived Motor Competence [39,40] or the Digital Scale of Perceived Motor Competence [41]. We included the PPC since this measure has been a commonly used assessment of perceived competence in this population [31,32,33] and was the outcome available to us in this secondary analysis. Still, future work should expand and replicate this work using different measures.

These results may also be explained by the differences between locomotor and ball skills themselves. Locomotor skills are more continuous and phylogenetic skills that commonly occur throughout a population. Children may choose to engage in locomotor skills when they have higher initial perceived competence regardless of environment. Comparatively, ball skills are more discrete and ontogenetic skills that are more specific to the individual. Children may be more encouraged to engage in ball skills based on factors other than perceived competence, such as motivation or instruction. There is limited evidence regarding how children engage in motor skill interventions [11,42]; hence how and why children engage in different types of skills remains unknown. Future research is warranted to examine children’s engagement in motor skill interventions and individual-level constraints that may predict this engagement and subsequent outcomes. This research is especially needed in gross motor skill interventions created using achievement goal theory and mastery climates such as CHAMP. Future research may be needed to determine how perceived competence relates to changes in other intervention climates; specifically low-autonomy intervention climates or climates not designed using achievement goal theory.

These results are also interesting from a conceptual perspective. There is moderate evidence to support a positive, reciprocal relationship between perceived competence and motor skills [38]. Still, the directionality of this relationship has not been well-established. Conceptual models posit that young children who cannot accurately perceive their abilities may exhibit inflated self-perceptions, which encourages them to engage in more skill practice resulting in improved motor skill competence [7]. The current findings only partially support perceived competence encouraging changes in young children’s motor skills. While PPC predicted changes in locomotor skills, the amount of variance explained was relatively small (approximately 7%), meaning that other factors appear to be driving children’s gains in motor skills across an intervention. More research is warranted to explore other factors, particularly individual constraints and psychosocial factors, that might relate to changes in skills across gross motor interventions.

Our results also found that sex moderated posttest ball skills but did not moderate posttest total motor skills or locomotor skills. These results indicate that, controlling for pretest and PPC, boys had higher posttest ball skills compared with girls. These findings are not surprising as there are established sex differences where boys outperform girls in the absence of an intervention [34,43,44], in certain skills after an intervention regardless of pretest skills [45] and after an intervention due to pre-existing differences in skill at pretest [46]. There are also reported sex differences in motor skills starting at the first year of life due to differences in parental promotion of play [47]. The importance of environments has also been suggested as a reason for existing sex differences in preschool-aged children [48]. These data add ongoing support to the role of sex in ball skills and suggest that girls might need additional support, environments, programs, or interventions to “catch up” to their male counterparts as early as 4 years of age.

This study included several strengths. First, this study used a documented and evidence-based 16-week CHAMP intervention [9,35], alongside frequently used and validated measures of both PPC [31,32,33] and motor skills [34,49]. PPC was also measured twenty-four hours before motor skills to ensure that children’s reported self-perceptions did not change based on earlier skill performance. This study also included several limitations. As previously mentioned, we only used one measure of perceived competence. This measure evaluated perceived physical competence, not specifically perceived motor competence. While this scale has been frequently used to evaluated perceived competence in young children with regards to their motor skills [31,32,33,50], the distinction between physical and motor competence is important to note when interpreting results. Utilizing multiple assessments of perceived competence will offer more insight into understanding if perceived competence predicts higher posttest scores after an intervention. Further, this scale is only validated for children over four and, while our sample had a mean age of four, a few children were between 3.5 and 4 years of age when they completed this assessment. Lastly, all children enrolled in the study attended Head Start Programs. Children enrolled in these schools come from families at or below the national poverty line, so it is unclear how these results may generalize to other populations.

## 5. Conclusions

Based on the importance of individual-level factors in performing motor skills [10], the purpose of this study was to examine if children’s PPC at the start of an intervention predicted changes in motor skills across an intervention. We were particularly interested in how PPC related to changes across an intervention grounded in the motivational theory of achievement goal theory. Results revealed that PPC was a significant predictor of change in locomotor and total skills but was not a significant predictor of change in ball skills across a mastery-oriented intervention (CHAMP). However, the variability explained by PPC was relatively low. These results are meaningful because they provide evidence that PPC may be an individual-level characteristic that predicts change in children’s motor skills, specifically in locomotor skills. However, additional work is needed to determine how other individual factors, particularly psychosocial factors, relate to children’s motor skill changes across interventions.

## Figures and Tables

**Table 1:** Efficacy of CHAMP intervention across TGMD scores.

	Pretest	Posttest	t	*p*
LM	14.66 ± 5.67	23.11 ± 7.15	11.37	<0.001
BS	16.03 ± 7.45	24.71 ± 8.21	11.77	<0.001
Total	30.82 ± 11.66	47.95 ± 13.75	15.28	<0.001

Note. LM = locomotor skills, BS = ball skill skills.

**Table 2:** Linear regression models predicting posttest motor skills.

		b	SE	*p*	^a^ R²
Total	Pretest	0.82	0.11	<0.001	0.59
	PPC	4.42	1.96	0.028	0.62
	Sex	1.43	2.34	0.542	0.62
LM	Pretest	0.67	0.13	<0.001	0.34
	PPC	3.20	1.23	0.012	0.41
	Sex	−1.57	1.42	0.275	0.43
BS	Pretest	0.63	0.10	<0.001	0.53
	PPC	2.01	1.21	0.10	0.54
	Sex	4.63	1.50	0.003	0.61

NOTE: LM = locomotor skills; BS = ball skills. ^a^ Due to the stepwise regression models, R^2^ values are presented for each sequential model.

## Data Availability

This project is a secondary data analysis from a larger research study. Once all analyses have been completed on the larger study, a final de-identified data set will be available upon request after signing a data usage agreement with the PI. Potential users of the data must agree to conditions of use, including but not limited to: restrictions against attempting to identify study participants, reporting responsibilities and proper acknowledgment of the data resource.
